# Surgical Turned-Downed CHIP Cases—Can PCI Save the Day?

**DOI:** 10.3389/fcvm.2022.872398

**Published:** 2022-04-07

**Authors:** Alexandru Achim, Madalin Marc, Zoltan Ruzsa

**Affiliations:** ^1^“Niculae Stancioiu” Heart Institute, University of Medicine and Pharmacy “Iuliu Hatieganu”, Cluj-Napoca, Romania; ^2^Klinik für Kardiologie, Medizinische Universitätsklinik, Kantonsspital Baselland, Liestal, Switzerland; ^3^Division of Invasive Cardiology, 2nd Department of Internal Medicine, University of Szeged, Szeged, Hungary

**Keywords:** multivessel disease, complex PCI, high risk, surgical ineligible, surgical turndown, CHIP, hemodynamic support devices

## Abstract

Current guidelines, rarely if at all, address decision-making for revascularization when bypass surgery is not a possibility for high-risk cases. Patients who are surgically turned down are routinely excluded from clinical trials, even though they remain symptomatic. Furthermore, the reasons for surgical ineligibility are often times not captured in standardized risk models. There is no data regarding health status outcomes following PCI procedures in these patients and the ultimate question remains whether the benefits of PCI outweigh its risks in this controversial subpopulation. When CHIP (Complex High risk Indicated Percutaneous coronary interventions) is selected for these very complex individuals, there is no unanimity regarding the goals for interventional revascularization (for instance, the ambition to achieve completeness of revascularization vs. more targeted or selective PCI). The recognition that, worldwide, these patients are becoming increasingly prevalent and increasingly commonplace in the cardiac catheterization labs, along with the momentum for more complex interventional procedures and expanding skillsets, gives us a timely opportunity to better examine the outcomes for these patients and inform clinical decision-making.

## Introduction

**Figure 1:**
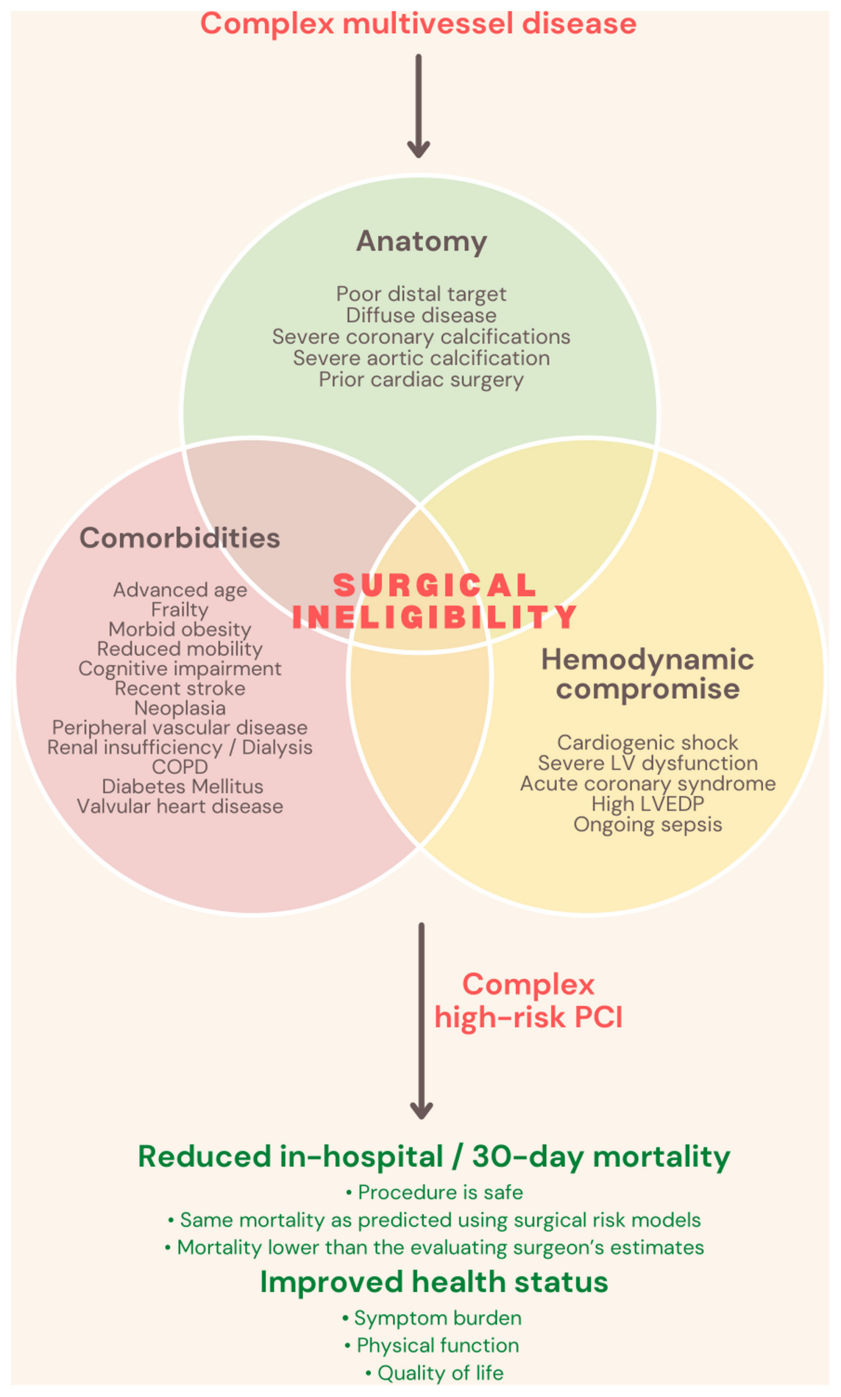
Complex high-risk indicated procedures definition and benefits. COPD, Chronic obstructive pulmonary disease; LV, left ventricle; LVEDP, left ventricular end-diastolic pressure; PCI, Percutaneous coronary intervention.

The proportion of percutaneous coronary intervention (PCI) to coronary artery bypass graft surgery (CABG) varies by nation. Still, it is commonly agreed that CABG is the revascularization technique of choice in the setting of left main disease (LMD) or multivessel disease (MVD) when clinically viable. This derived on account of randomized controlled trials which compared revascularization strategies in MVD and found that CABG is associated with fewer repeat revascularization procedures and improved survival (1, 2), even if PCI is performed using the latest generation drug-eluting stents and is guided by fractional flow reserve (FFR) (3–5). Nonetheless, in medical practice, physicians frequently encounter patients who would have been excluded from clinical trials because of significant medical comorbidities. In such patients, CABG and thus the findings of these trials are not applicable. As a consequence, the undisputed performance of CABG in LM and MVD decreases in front of frail patients with multiple comorbidities. With an aging patient population, a growing challenge remains the management of these patients, with severe ischemic heart disease. Comorbidities increase the patient's surgical risk and can negate the benefits of surgical revascularization, around one in five patients with left main and/or multivessel disease being declared surgically ineligible (6). Current guidelines rarely, if at all, address decision-making for revascularization when bypass surgery is not a possibility, and patients who are surgically turned down are routinely excluded from clinical trials, even though they remain symptomatic. Further, the reasons for surgical ineligibility are seldom captured in standardized risk models. There is no existing data regarding health status outcomes following PCI procedures in such patients, and the ultimate question remains whether the benefits of PCI outweigh its risks in this subpopulation. It should not be forgotten that this topic addresses a particular category of patients: mostly octogenarians, with multiple comorbidities, fragile, some with a history of neoplastic disease, some with reduced mobility and a survival less than a year. Nonetheless, with revascularization, both survival and quality of life can increase (Figure 1).

Finally, when CHIP (Complex High risk Indicated Percutaneous coronary interventions) is selected for these very complex individuals, there is no unanimity regarding the goals for interventional revascularization (for instance, the ambition to achieve completeness of revascularization vs. more targeted or selective PCI). The recognition that, worldwide, these patients are becoming increasingly prevalent and increasingly commonplace in the cardiac catheterization labs, along with the momentum for more complex interventional procedures and expanding skillsets, gives us a timely opportunity to better examine the outcomes for these patients and inform clinical decision-making.

## The Optimum Trial—Review of Results

The OPTIMUM study (*clinicaltrials.gov identifier: NCT02996877*), the first to investigate this category of patients, was an investigator-initiated prospective multicenter study conducted at 22 centers in the United States. It included up to 750 patients who, after evaluation, were deemed by the site heart team (comprised of both an interventional cardiologist and a cardiothoracic surgeon) to be unsuitable for surgery. Of these, 726 underwent PCI, while 24 were assigned to medical therapy. The outcomes were presented at the Transcatheter Cardiovascular Therapeutics 2021 Scientific Sessions (7).

The baseline characteristics of the study cohort imply a very high-risk population with complex disease and associated comorbidities. Most of the patients were over the age of 70, and 31.5% were female. At baseline, more than half (56.6%) of the patients were diagnosed with diabetes mellitus, while 48.2% had a history of myocardial infarction, and 32.8% had received prior PCI. Other traits included prior CABG (16.4%), current smoking (18.2%), renal failure (37.2%), atrial fibrillation (23.1%), and New York Heart Association (NYHA) class III/IV heart failure (23.4%). The heart team rated them as high-risk for the following reasons: 16.8% had severe left ventricular dysfunction or non-viable myocardium, 18.9% had poor distal targets, 16.8% had advanced lung disease, and 10.1% were reportedly frail and/or advanced in age. The most common reason for revascularization was stable or unstable angina. Not only was the patient population at high risk, but the coronary anatomy was complex: 80% of patients had severe calcification, bifurcation disease, and lesions >20 mm in length. The average SYNTAX score was 32.4, with 45.3% of patients having a high SYNTAX score (≥33). Chronic total occlusions were frequent (57.0%), and LMD cases were not uncommon either (38.2%). Mechanical circulatory support (MCS) was used in 27% of the cases—contrary to popular belief, the enrolled American centers were not excessive of this. Furthermore, unlike other studies testing for PCI performance, intravascular imaging was often used (63.9%). Complications were reported in 9.8% of the cases.

The primary end-point was to compare the 30-day and in-hospital mortality in the PCI cohort with the predicted Society of Thoracic Surgeons (STS) surgical risk. For the secondary objective, the investigators analyzed and compared the 30-day and in-hospital mortality in the PCI cohort with the EuroSCORE II and the Surgeon's predicted risk. The results were also compared according to the level of completeness of the revascularization. Another important aspect was the quality of life of these patients, and this was assessed using two questionnaires: the Seattle Angina Questionnaire (quality of life) and the Kansas City Cardiomyopathy Questionnaire (angina frequency and heart failure).

The observed death rate at 30 days was 5.6%, which matched the predicted risk of death using the STS and EuroSCORE II risk calculators (5.3 and 5.7%, respectively). On the other hand, the site surgeon's predicted mortality was 10.4%. The actual death rate was 40% lower than that predicted by the site surgeons, at least with surgery. Tellingly, however, by 6 months, the mortality more than doubled in the PCI cohort (12.3%).

At 6 months, the investigators detected significant improvements in patient-reported health status amongst the survivors, including marked improvements in quality of life and reductions in angina frequency. More than 82% of patients had no angina at 6 months compared with 40.5% at baseline, while 11.6% reported monthly episodes, down from 31.9% at baseline. In total, 6% reported weekly or daily angina, which was down from 27.7% before PCI.

## Should Mortality be the Only Point of Focus?

Given the overestimation of risk during the CABG rejection decision, the inevitable question is whether these patients should be reconsidered for surgery. OPTIMUM suggests that the outcomes are similar to the current risk models and appreciably lower than surgeons' assessments. Caution should be taken as the STS and EuroSCORE II scores were intended to assess surgical mortality, and OPTIMUM looked at the relationship to PCI-related mortality. Moreover, in such a sickly population, it would be misleading to expect that the actual surgical mortality rates would be exactly what the STS and EuroSCORE II risk scores predict. Perhaps one interpretation might be that PCI mitigates the risk anticipated by the surgeons given the lack of periprocedural morbidity and complications associated with invasive surgery. Lastly, STS and EuroSCORE II do not capture all of the risk characteristics that impact a surgeon's reasons for turning down patients (for example, poor distal target/conduit, non-viable myocardium, obesity, prior stroke).

The concept of CHIP remains somewhat ill-defined with considerable variability among operators, making it difficult to delineate the difficulties of such a procedure and how they may be related to prognosis. A recent multiple logistic regression model of a large British population found 7 patient factors (age >80 years, female sex, previous stroke, previous myocardial infarction, peripheral vascular disease, ejection fraction <30%, and chronic renal disease) and 6 procedural factors (rotational atherectomy, left main PCI, 3-vessel PCI, dual arterial access, MCS, and total lesion length >60 mm) associated with increased in-hospital major adverse cardiac and cerebrovascular events (MACCE) and attempted to construct a CHIP score (8). Interestingly, MCS had the strongest association with MACCE. Even though MCS aimes to reduce MACCE, we concur with the investigators that the increased risk reported is mainly related to the fact that LV support is preferentially used in patients with an intrinsically high-risk profile. Indeed, CHIP is closely related to MCS, similar to those of the OPTIMUM patients were recruited in the MCS studies (9–11). In addition to their main results, all advocating for supported PCI, PROTECT II (9), BCIS-1 (10) and the Roma-Verona Registry (11) univocally found a significant increase in LVEF and a significant improvement in functional status after revascularization. Although guidelines support the use of mechanical LV support during high-risk PCI (12, 13), the observed low rate of planned MSC use in OPTIMUM or other large CHIP registries (8) could be explained by the increasing operator comfort in CHIP over time, a lack of robust clinical data supporting their use, cost, concerns regarding the safety and morbidity of the devices themselves, and, of course, the ambiguous definition of CHIP that we mentioned earlier.

Because more than half of the OPTIMUM patients were elective (stable angina or atypical angina), in light of the results of the ISCHEMIA trial (14, 15), one could argue why these patients cannot remain on medical therapy. As aforementioned, it is crucial to analyze what type of population these results can be applied to. Among the exclusion criteria of the ISCHEMIA trial, we mention left ventricle ejection fraction <35%, NYHA class III-IV heart failure, exacerbation of chronic heart failure within the previous 6 months, LM stenosis, prior CABG, recent acute coronary syndrome, recent stroke, estimated glomerular filtration rate <30 mL/min, severe valvular disease, and life expectancy <5 years. This criterion is similar to the type of patients recruited into OPTIMUM. This would make the plea for conservative treatment in these patients inappropriate. OPTIMUM did not randomize 1:1 with medical treatment, due to the short follow-up limitation and the variety of comorbidities and anatomical complexity in this population. In a similar study, Graham et al. managed to cast a glance at this detail, demonstrating that elderly patients with ischemic heart disease who underwent revascularization with either PCI or CABG had better outcomes at 4 years than those treated with medication alone (16). However, given the improvements in techniques for PCI, most patients turned down for surgery will undergo PCI, thus, the number of patients with MVD treated medically who are ineligible for CABG is likely quite small. It was also the case with OPTIMUM, where initially, the investigators intended to include a group of patients who had no revascularization options, namely patients treated with medical therapy alone. Still, given the increasing prevalence of PCI patients, they later modified the protocol to include only those patients who underwent CHIP.

In a retrospective analysis from 2008 to 2012, Danson et al. showed that in a rather morbid population, the MACCE rate at 30 days is similar between the group treated with PCI and the group treated with medical therapy alone. However, after 1 year, MACCE were significantly higher in the medical treatment group (17). Furthermore, the residual SYNTAX score (an index of incomplete revascularization) was independently associated with MACCE at 1 year. The fact that in-hospital mortality did not increase in the PCI group, along with the long-term outcomes, supports the hypothesis that PCI with complete revascularization may confer the greatest predicted benefit from revascularization. Indeed, OPTIMUM and the Roma-Verona Registry also noted a trend toward better in-hospital/30-day mortality, left ventricle ejection fraction and 6-month health status improvement in those with a lower residual SYNTAX score (7, 11).

Shield et al. have similarly focused on patients with advanced CAD who were deemed to be ineligible for surgery, retrospectively reviewing a smaller cohort (137 patients) and showing even better results for PCI (mortality 2.2% at 30 days and 11% at 1 year) but in a healthier population (Syntax Score >33 pcts in 14% of patients vs 45% in OPTIMUM, STS >8% only 17%) (18). It is not surprising that mortality increases with the level of comorbidity but also with the complexity of the coronary disease. On the other hand, the operator cannot influence the first factor, but mechanical support, debulking devices, less iodine-contrast, full revascularization, or centers with experience in CHIP are all aspects which can make the risk of the procedure go down (19). The SYNTAX trial included a nested registry of patients ineligible for surgery who were treated with PCI (20). Among those patients, the EuroSCORE II was 5.8%, similar to that of OPTIMUM. At 30 days, the rate of all-cause mortality was 3.1%. A 10-year follow-up in these patients would be interesting to see, although it must be clearly acknowledged that at the time of enrollment in the SYNTAX, most patients were over 70 years old, so we should not be surprised at a possible mortality of 50% at present. Maybe mortality alone should not be our only point of focus in trials that test the performance of PCI in general, but especially in this old, fragile population of patients who are already living with low life expectancy, but in whom, through PCI, the quality of life is improved.

At 6 months, the mortality rate more than doubled, reflecting the high-risk nature of this population, but the risks (compared to those calculated by STS and EuroSCORE II) of the intervention did not exceed the net benefits in terms of significant improvement in patients' reported health status. Should we be doing PCI in high-risk patients with 30-day mortality following PCI, which is around 5–6%? We learn from this study that marked improvement in severe angina and quality of life can be achieved (at 6 months, 80% of patients had no angina, 11% had monthly residual angina only). We can immediately see that, effectively, for over 90% of our patients, we are reducing the symptom burden to less than once a month. This is a crucial aspect as, in randomized controlled trials, our pivotal objective addresses only if “there is a mortality benefit in these patients.” Still, we must acknowledge that, often in this particular morbid population, it is unlikely we are going to impact on their longer-term prognosis, but the quality of life and symptoms are still important to patients, and the sight of that should not be lost. OPTIMUM and other registries of its kind (6, 9–11, 16, 18) show that we should reflect on the patients' cohort that we are undertaking these procedures on and think about what really matters to them.

Needless to say, we consider the 6-month data from OPTIMUM preliminary as the investigators will have to wait for the 1-year results. As Table 1 shows, a major limitation that reigns over this controversial population is the lack of data on intermediate and long-term outcomes. In the last 10 years, 6 studies have been found describing outcomes in patients undergoing PCI who have been turned down for CABG on the basis of prohibitive risk. Of these, Sukul et al.'s criteria for surgical ineligibility may have been biased due to a lack of clear referral documentation and how patients were extracted from the registry, hence, their much lower event rate (Table 1).

**Table 1:** Distribution of percutaneously treated coronary artery disease in surgical turndowned patients, comorbidity and anatomical stratification−6 studies across 10 years.

**Study**	**McNulty et al. (21)**	**Danson et al. (22)**	**Waldo et al. (6)**	**Sukul et al. (23)**	**Danson et al. (17)**	**Shields et al. (18)**	**OPTIMUM (7)**
**Year**	**2011**	**2014**	**2014**	**2016**	**2018**	**2020**	**2021**
Study design	Retrospective, single-center	Retrospective, single-center	Retrospective, multicenter	Retrospective, multicenter	Retrospective, multicenter	Retrospective, single-center	Prospective, multicenter
Number of patients	55	77	218	1922	133	137	750
Age (years)	75 ± 10	74 ± 1.2	72 ± 12	64.5 ± 11.8	76 ± 9	71 ± 11.1	70.0 ± 10.9
At least 5 comorbidities	55%	–	44.6%	28.4%	49.7%	45.8%	31.9%
LVEF	45 ± 17%	–	–	53.6 ± 12.0%	–	44.3 ± 15.1%	42.6 ± 16.3%
LM PCI	100%	–	33%	1%	45.8%	40%	38.2%
High SYNTAX score (>33 pcts)	39%	–	41%	8.4%	43.5%	14%	45.3%
ACS presentation	62%	–	22%	24.3%	58%	–	37.7%
30 day MACCE	3.6%	6 ± 1.1%	7%	0.83%	12.2%	2.9%	5.6%
6 months MACCE	–	–	–	–	–	–	12.3%
1 year MACCE	–	22 ± 1.9%	–	–	26.7%	27.7%	–

*Where nominal values are used, they are presented as mean standard deviation*.

*LVEF, left ventricle ejection fraction; LM, left main; PCI, percutaneous coronary intervention; ACS, acute coronary syndrome; MACE, major adverse cardiac and cerebrovascular events*.

## Perspectives

The reported lower frequency of angina, improvement in overall quality of life and reduction in in-hospital/30-day mortality rates suggest there is room for high-risk PCI in patients with no other options and that this procedure is in fact safe. The potential of CHIP has changed significantly for the better in recent years, and the credit goes both to technological progress and to the tertiary, high-volume centers that have trained skilled operators in this regard. There is no data comparing the difference in outcomes between centers of expertise and medium-volume centers when performing CHIP, but it can be clearly seen that the OPTIMUM cohort represents tough cases/complex patients and a collateral finding from OPTIMUM which provokes the reader is where and who should do these procedures. Currently, CHIP can be performed, but the operators must be circumspect and judicious. The safety of the procedure and its outcome can only be improved by the decision of the Heart Team. Postoperative care should not be neglected. The cause of early mortality has not been revealed, but surely factors such as contrast-induced nephrotoxicity or sepsis can negatively affect it.

Further studies are needed to assess technical considerations in the surgical turndown population; such issues include the impact of completeness of revascularization, the value of MCS for safer and optimal revascularization, and even the possibility of very short dual antiplatelet or single antiplatelet therapy. And of course, a question remains whether this single study is sufficient to change guidelines to include CHIP for patients with prohibitive risk.

## Author Contributions

AA, MM, and ZR contributed to the conception and design of the study. AA organized the database and wrote the first draft of the manuscript. MM and ZR wrote sections of the manuscript. All authors contributed to manuscript revision, read, and approved the submitted version.

## Conflict of Interest

The authors declare that the research was conducted in the absence of any commercial or financial relationships that could be construed as a potential conflict of interest.

## Publisher's Note

All claims expressed in this article are solely those of the authors and do not necessarily represent those of their affiliated organizations, or those of the publisher, the editors and the reviewers. Any product that may be evaluated in this article, or claim that may be made by its manufacturer, is not guaranteed or endorsed by the publisher.

## Abbreviations

PCI: percutaneous coronary intervention
CABG: coronary artery bypass grafting
LMD: left main disease
MVD: multivessel disease
FFR: fractional flow reserve
CHIP: complex high risk indicated PCI
NYHA: New York Heart association
MCS: mechanical circulatory support
STS: Society of Thoracic Surgeons
MACCE: major adverse cardiac and cerebrovascular events.
